# Impact of the COVID-19 pandemic on perinatal mental health screening, illness and pregnancy outcomes: A cohort study

**DOI:** 10.1177/1753495X221139565

**Published:** 2022-11-28

**Authors:** Andre C. Q. Lo, Michelle Kemp, Nikolett Kabacs

**Affiliations:** 1 School of Clinical Medicine, University of Cambridge, Cambridge, UK; 2 Department of Obstetrics and Gynaecology, 2153 Cambridge University Hospitals NHS Foundation Trust, Cambridge, UK; 3 Perinatal Mental Health Team, 4028 Cambridgeshire and Peterborough NHS Foundation Trust, Fulbourn, UK

**Keywords:** Pregnancy, postpartum depression, mental health, COVID-19, pandemic

## Abstract

**Background:**

The aim was to explore the impact of the COVID-19 pandemic on perinatal mental health screening, illness and related pregnancy complications/outcomes.

**Methods:**

A single-centre retrospective cohort study in mothers giving birth before versus during the pandemic. Primary outcomes were the comparative prevalence/incidence of peripartum psychiatric diagnoses. Secondary outcomes were the pandemic's effect on psychiatric screening accuracy, and on other pregnancy outcomes linked to mental health.

**Results:**

The pandemic did not significantly increase the crude incidence of diagnosed peripartum anxiety (risk ratio (RR) = 1.39, 95% CI = 0.66–2.95), depression (RR = 1.63, 95% CI = 0.72–3.70) or other pregnancy outcomes. In multivariate models, the pandemic decreased Apgar scores and was involved in interaction effects for postpartum mental illness and birthweight. Psychiatric screening at the booking appointment exhibited lower sensitivity in predicting antenatal mental illness (pre-pandemic = 85.71%, pandemic = 25.00%; *p* = 0.035).

**Conclusions:**

The lowered screening sensitivity likely meant mental illness was poorly anticipated/under-detected during the pandemic, leading to no crude increase in perinatal psychiatric diagnoses.

## Background

The COVID-19 pandemic has brought with it a myriad of stressors, from socially isolating public health measures to financial difficulties and barriers in accessing healthcare. With the perinatal period being a physically and emotionally challenging time, pregnant and postpartum individuals are especially vulnerable to the psychosocial effects of the pandemic. A meta-analysis found a high prevalence of anxiety and depression among pregnant women during the pandemic, respectively, 56% (95% CI: 28–85) and 39% (95% CI: 19–59) in Europe.^1^ In comparison, pre-pandemic meta-analyses estimated the prevalence of the disorders at 13.4% (95% CI: 8.2–18.7) and 9.2% (95% CI: 8.4–10.0), respectively, in high-income countries.^2,3^

Mental health difficulties are underrecognized and undertreated in the perinatal period,^4,5^ so it is unclear if the noted deterioration in mental health has translated to an increased presentation of perinatal psychiatric disorders to health services to the degree indicated by these meta-analyses. This is especially true given the disruption to obstetric health service provision and access caused by the pandemic with ambiguous ramifications for the screening, detection and treatment of perinatal mental illness.

Furthermore, perinatal mental disorders are detrimental to physical health, being associated with adverse pregnancy outcomes and complications including hypertensive disorders of pregnancy, gestational diabetes, preterm birth, low birth weight and lower Apgar scores.^1^ The incidence of low birth weight and hypertensive disorders of pregnancy were reported to have decreased during the pandemic,^6,7^ but it is uncertain how these changes may be related to interactions between the pandemic and mental health.

The primary aim of this study was to determine whether the prevalence/incidence of perinatal psychiatric disorders diagnosed in routine practice was elevated during the COVID-19 pandemic in line with what has been reported on the pandemic's impact. Secondary objectives included examining if the observed pattern of psychiatric diagnoses was associated with pandemic-related changes to mental health screening, as well as if any relationship between the pandemic and perinatal mental health had potential effects on pregnancy complications and outcomes.

## Methods

### Aims and objectives

A cohort study was conducted on mothers who gave birth before and during the COVID-19 pandemic to examine the effect of the pandemic on perinatal mental health screening and diagnoses, along with potential relationships to pregnancy complications/outcomes.

The primary outcomes were the risk of peripartum anxiety or depression diagnosed during versus before the pandemic (based on medical notes). A number of secondary outcomes were investigated as follows. At our institution, it is intended that midwives perform mental health screening using a five-question tool, which includes the Whooley questions and a comments section (Supplementary Appendix 1) at the booking appointment (first antenatal appointment with a midwife).^8^ Besides examining screening coverage, the sensitivity and specificity of this tool to predict antenatal psychiatric disorders (using documented perinatal mental illness in the medical notes as the reference standard) were assessed to provide context for the observed rates of perinatal mental illness diagnoses, especially given this screening was conducted almost entirely by telephone instead of in person during the pandemic. Changes to pregnancy outcomes known to be linked to perinatal mental health were also explored. In addition, crude analyses were re-evaluated using multivariate models to re-examine associations after controlling for potential confounders.

### Enrolment and data collection

The medical notes of all mothers giving birth from 23 to 29 November 2019 (pre-pandemic) or 23 to 29 November 2020 (pandemic) under the care of a UK tertiary hospital were retrospectively reviewed for diagnoses, complications and outcomes. This timing meant that the latter cohort delivered during the second national lockdown (5 November to 1 December 2020 in response to the coronavirus second wave), whilst having had their booking appointment after 23 March 2020, the start of the UK's first COVID-19 lockdown.

Only clinical data collected during routine clinical care was used in this project; service evaluation was locally approved under the registration number PRN10177. As per the Governance Arrangements for Research Ethics Committees, research using only previously collected, non-identifiable data requires no ethical review.^9^

### Definition of outcomes

Although the perinatal period can be defined as including pregnancy and one year postpartum, there was an overlap in the pre-pandemic cohort of the first postpartum year with the start of the pandemic. Hence for this study, postpartum was defined as the first four weeks following delivery in line with the Diagnostic and Statistical Manual of Mental Disorders, Fifth Edition (DSM-5) demarcation^10^; accordingly, peripartum meant during pregnancy or the first four postpartum weeks.

For incidence, the onset of symptoms for a mental health episode had to be deemed to have occurred during the relevant time frame (during pregnancy for antepartum disorders, within four weeks for postpartum disorders, with peripartum disorders encompassing both antepartum and postpartum periods). On the other hand, prevalence estimates included ongoing treatment for a mental health episode with onset prior to the specified time frame. Post-traumatic stress disorder and phobias were classified as anxiety disorders.

### Statistical analysis

The impact of the pandemic on psychiatric diagnoses was examined primarily via crude risk ratios (RRs) and secondarily in multivariate logistic regressions. As records pertaining to the postpartum period were primarily covered by primary care records, subjects whose primary care records were unavailable were excluded from analyses of postpartum mental illness. Similarly, the impact of the pandemic on pregnancy complications or outcomes was examined using crude analyses and multivariate logistic or linear regressions. Apgar scores were reversed (i.e. Apgar score of 1 was coded as 10) and analysed using a log link Gamma model instead of the conventional Gaussian linear regression model.

For crude analyses, student's *t*-tests (or Mann–Whitney *U* tests for non-normal data) and chi-squared tests (or Fisher's exact tests if counts ≤5) were used to compute *p*-values for continuous and binary data, respectively. For multivariate models, besides including the pre- or intra-pandemic timing of pregnancy as a covariate, variables were included if they were likely to confound associations based on the literature. In general, all multivariate models included baseline characteristics as covariates, and regressions examining postpartum outcomes further included pregnancy characteristics as covariates. Socioeconomic status was assessed using the English Index of Multiple Deprivation 2019 deciles, the official national measure of relative deprivation which ranks postcodes based on seven domains: income, employment, education, health, crime, housing/service barriers and living environment (decile 1 is the most deprived 10%, decile 10 is least deprived 10%).^11^ As it was possible that the pandemic and psychiatric disorders disproportionately affect specific subgroups, interaction effects involving the pandemic (e.g. between pandemic and age) or antenatal mental illness were examined and included in the models where appropriate. As a sensitivity analysis, we produced parsimonious models using backwards stepwise regression to examine how effect estimates varied based on model assumptions.

A sample size calculation indicated that a recruitment of 58 subjects per comparison group would allow for the detection, at a power of 0.95 and significance level of 0.05, of an anticipated difference in antenatal depression prevalence of approximately 9.2% versus 39% before and during the pandemic.^1,2^

All tests were two-tailed with a significance level set at 0.05. Analyses were conducted in OpenEpi and R (Version 4.0.5).^12,13^

## Results

### Cohort characteristics

In total, 191 mothers, 92 delivering before and 99 delivering during the pandemic, were included in this study. Baseline characteristics of age and ethnicity were not significantly different between the pre-pandemic and pandemic populations (Table 1). Mothers delivering during the pandemic lived in higher socioeconomic areas based on the 2019 Index of Multiple Deprivation (*p* = 0.008) were more likely to have a psychiatric history (*p* = 0.047) and were more likely to have higher gravidity (*p* = 0.030) and parity (*p* = 0.047).

**Table 1.** Characteristics of the pre-pandemic and pandemic cohorts.

Variables	Pre-pandemic (*n* = 92)			Pandemic (*n* = 99)			*p*-value
	*n*	*Mean*	*SD*	*n*	*Mean*	*SD*	
Age at delivery	92	31.75	4.80	99	32.97	5.16	0.091
Ethnicity	58 White (75.32%), 19 Non-white/mixed			81 White (86.17%), 13 Non-white/mixed			0.070
	15 unknown			5 unknown			–
IMD (decile)	92	6.73	2.17	99	7.57	2.05	0.008
Gravidity	92	2.15	1.55	99	2.40	1.33	0.030
Parity	92	0.76	1.073	99	0.96	1.01	0.047
Nulliparity	48 (52.17%)			37 (37.37%)			0.040
Psychiatric history	23 (25.00%)			38 (38.38%)			0.047
Obesity affecting pregnancy	12 (13.04%)			12 (12.12%)			0.848

IMD: index of multiple deprivation (lower values indicate increased deprivation).

### Risk of psychiatric diagnoses

For our primary outcomes, the incidence of peripartum psychiatric diagnoses was not significantly elevated compared to previously in unadjusted analyses (20.20% vs. 16.30%; RR = 1.24, 95% CI = 0.68–2.27), nor was any association found in prevalence analyses or subgroup analyses examining depression, anxiety and the antepartum or postpartum periods separately (Table 2).

**Table 2.** Changes to psychiatric outcomes before and during the COVID-19 pandemic.

Variables	Pre-pandemic (*n* = 92 total, *n* = 87 for postpartum)	Pandemic (*n* = 99 total, *n* = 77 for postpartum)	Crude risk ratio (95% CI)
Incidence			
Peripartum psychiatric disorder	15 total (16.30%)	20 total (20.20%)	1.24 (0.68–2.27)
	10 antepartum (10.87%)	13 antepartum (13.13%)	1.21 (0.56–2.62)
	8 postpartum (8.70%)	12 postpartum (12.12%)	1.33 (0.57–3.08)
Peripartum depression	8 total (8.70%)	14 total (14.14%)	1.63 (0.72–3.70)
	5 antepartum (5.43%)	7 antepartum (7.07%)	1.30 (0.43–3.96)
	5 postpartum (5.43%)	10 postpartum (10.10%)	1.77 (0.63–4.95)
Peripartum anxiety	10 total (10.87%)	15 total (15.15%)	1.39 (0.66–2.95)
	8 antepartum (8.70%)	9 antepartum (9.09%)	0.85 (0.40–1.84)
	4 postpartum (4.35%)	8 postpartum (8.08%)	1.77 (0.55–5.65)
Postpartum psychosis	1 (1.09%)	1 (1.01%)	0.89 (0.06–3.91)
Prevalence			
Peripartum psychiatric disorder	18 total (19.57%)	22 total (22.22%)	1.14 (0.65–1.98)
	15 antepartum (16.30%)	15 antepartum (15.15%)	0.93 (0.48–1.79)
	14 postpartum (15.22%)	15 postpartum (15.15%)	0.95 (0.49–1.64)
Peripartum depression	12 total (13.04%)	16 total (16.16%)	1.24 (0.62–2.48)
	9 antepartum (9.78%)	10 antepartum (10.10%)	1.03 (0.44–2.43)
	10 postpartum (10.87%)	12 postpartum (12.12%)	1.06 (0.49–2.32)
Peripartum anxiety	14 total (15.22%)	17 total (17.17%)	1.13 (0.59–2.16)
	12 antepartum (13.04%)	11 antepartum (11.11%)	0.85 (0.40–1.84)
	10 postpartum (10.87%)	11 postpartum (11.11%)	0.97 (0.44–2.17)

**Figure 1.**
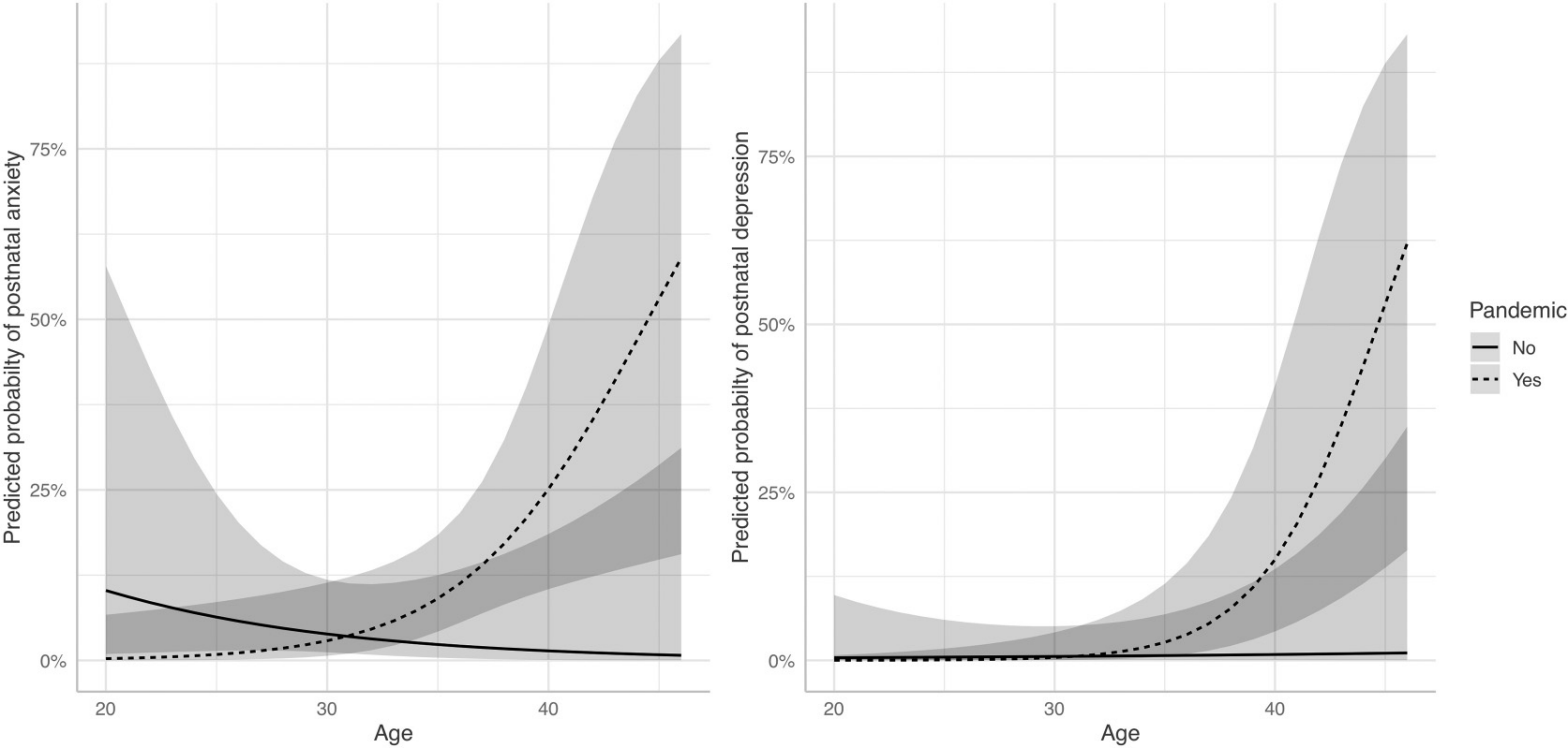
Interaction plots between the pandemic with age and risk of postnatal anxiety (left) or postnatal depression (right) from parsimonious models with 95% confidence intervals.

In multivariate analyses of incidence (Table 3), no association between the pandemic and antenatal psychiatric diagnoses was found, but postnatally there was a significant interaction between the pandemic and age, with an elevated risk of postpartum anxiety and depression in older mothers only during the pandemic (*p* = 0.010 and 0.024, respectively) (Figure 1). Antenatal anxiety was also significantly associated with lower age, increased gravidity and having a psychiatric history; meanwhile, depression was associated with psychiatric history and obesity antenatally, and psychiatric history, parity and antenatal anxiety postnatally. Associations observed in these full multivariate models were largely consistent with those from parsimonious models (Supplemental Table 1).

**Table 3.** Association of covariates to the incidence of psychiatric outcomes in multivariate models.

Variable	OR (95% CI)			
	Anxiety		Depression	
	Antepartum (*n* = 171)	Postpartum (*n* = 146)	Antepartum (*n* = 171)	Postpartum (*n* = 146)
Pandemic	1.00 (0.31–3.37)	0 (0–0.03)	1.17 (0.31–4.74)	0 (0–0.10)
Age at delivery (years)	0.88 (0.78–0.99)	0.84 (0.64–1.08)	0.92 (0.80–1.05)	0.97 (0.74–1.26)
White ethnicity	2.41 (0.39–47.05)	1.04 (0.10–24.45)	1.49 (0.22–30.51)	0.18 (0.02–1.58)
IMD (decile)	0.91 (0.67–1.22)	0.77 (0.54–1.07)	0.91 (0.65–1.29)	0.86 (0.59–1.24)
Gravidity – Parity	2.47 (1.16–5.44)	1.06 (0.36–2.83)	1.41 (0.54–3.35)	0.74 (0.26–1.87)
Parity	0.75 (0.37–1.30)	1.07 (0.50–2.13)	0.87 (0.44–1.50)	2.09 (1.00–4.72)
Psychiatric history	5.20 (1.58–19.79)	2.25 (0.42–12.31)	15.50 (3.40–119.93)	8.65 (1.59–63.69)
Obesity affecting pregnancy	2.60 (0.50–11.36)	7.46 (0.89–67.70)	7.06 (1.37–38.87)	2.18 (0.17–29.07)
Hypertension in pregnancy	–	0 (0–8.35 × 10^43^)	–	0 (0–4.65 × 10^41^)
Diabetes/ Gestational diabetes	–	2.53 (0.24–21.61)	–	1.24 (0.08–14.08)
Estimated blood loss at delivery (L)	–	0.68 (0.07–3.92)	–	3.61 (0.67–19.33)
Antenatal depression	–	0.72 (0.06–7.11)	–	1.93 (0.29–12.78)
Antenatal anxiety	–	6.65 (0.71–74.00)	–	28.83 (3.27–407.11)
Age × pandemic (interaction)	–	1.60 (1.14–2.34)	–	1.54 (1.10–2.35)

IMD: index of multiple deprivation (lower values indicate increased deprivation).

### Accuracy of mental health screening

Before the pandemic, any mental health difficulty noted in the screening tool used at the booking appointment (including those only documented in the comments section of the tool) predicted having a psychiatric disorder affecting the antenatal period with 85.71% (95% CI: 48.69%–97.43%) sensitivity and 89.36% (95% CI: 77.41%–95.37%) specificity (Table 4). In comparison, screening during the pandemic was almost always via telephone instead of in-person and exhibited a significantly lower sensitivity (*p* = 0.035) of 25.00% (95% CI: 8.89%–53.23%) but comparable (*p* > 0.999) specificity of 89.83% (95% CI: 79.54%–95.26%).

**Table 4.** Sensitivity and specificity of psychiatric screening tool at booking an appointment in predicting antepartum psychiatric disorder.

Screening	Pre-pandemic (*n* = 54)		Pandemic (*n* = 70)	
	Disorder	No disorder	Disorder	No disorder
Positive screen	6	5	3	6
Negative screen	1	42	9	53
	Sensitivity	Specificity	Sensitivity	Specificity
	85.71%	89.36%	25.00%	89.83%

Rates of mental health screening using this tool at the booking appointment did not drop during the pandemic compared to previously (97.30% vs. 88.52%, *p* = 0.099) (Table 5). Around a third of all patients did not have details of their booking appointment documented because it was conducted out of the area in other institutions. Mental health screening using a tool at the 6-week postnatal appointment was rarer in comparison, being documented four times in each cohort where the Patient Health Questionnaire-9 or Edinburgh postnatal depression scale was used. Other than at these two appointments, formal screening for mental illness was not regularly performed.

**Table 5.** Changes to psychiatric screening before and during the COVID-19 pandemic.

Variables	Pre-pandemic (*n* = 92 total, *n* = 87 for postpartum)	Pandemic (*n* = 99 total, n = 77 for postpartum)	*p*-value
Psychiatric screening using a tool at booking appointment	54 screened (88.52%), 7 unscreened	70 screened (97.30%), 2 unscreened	0.099
	31 unknown^a^ (33.70%)	27 unknown^a^ (29.29%)	–
Psychiatric screening using a tool at 6-week postnatal appointment	3 PHQ-9, 1 EPDS (4.35% total)	4 EPDS (4.04%)	>0.999

PHQ: Patient Health Questionnaire; EPDS: Edinburgh Postnatal Depression Scale.

^a^Details of booking appointment were not documented (e.g. booking occurred out of area).

### Risk of other pregnancy outcomes

In unadjusted analyses, pregnancy complications and outcomes did not differ significantly between the pre-pandemic and pandemic cohorts (Supplemental Table 2). In multivariate analyses examining the combined pre-pandemic and pandemic cohorts, antenatal anxiety was associated with decreased gestational age (β = −6.09 days, 95% CI = −11.81 to 0.36); in turn, gestational age predicted birth weight (β = 24.00 g/day, 95% CI = 17.57–30.42) thereby mediating the effect of antenatal anxiety on birth weight. Birth weight was further affected by interaction effects where hypertensive patients had offspring with lower birth weight during the pandemic only (*p* = 0.039), as well as where those with antenatal depression had offspring with lower birthweight if they lived in more deprived neighbourhoods (*p* = 0.014) (Supplemental Figure 1). The pandemic was also associated with lower Apgar scores at 1 min (16.08% average increase in the reverse Apgar score, 95% CI = 0.58%–33.89%), which in turn predicted Apgar at 5 min (25.42% average increase in the reverse 5-min Apgar score/unit increase in 1-min Apgar score, 95% CI = 20.37%–30.79%). No evidence of interaction effects between the pandemic and antenatal psychiatric illness for\ maternal/neonatal outcomes was observed.

## Discussion

This study did not find a significantly increased crude risk of peripartum anxiety or depression diagnoses during the pandemic as one would expect from the literature. This is the first study to our knowledge that examined and found that the pandemic may have adversely affected the ability of psychiatric screening at the initial antenatal appointment to predict if mental health issues will affect pregnancy. The pandemic may have also altered risk patterns for perinatal outcomes, with an elevated risk of postpartum depression or anxiety for older women during the pandemic. Although some effect of the pandemic on Apgar scores and birthweight was observed, there was no evidence of interaction effects between the pandemic and antenatal mental illness for maternal or neonatal outcomes; the lack of an association between the pandemic and antenatal psychiatric diagnoses also precluded mediation effects (where mental health may have mediated potential effects of the pandemic on perinatal outcomes).

Since formal mental health screening is not undertaken regularly throughout pregnancy in many cases, it is important to recognise that psychiatric screening tools are utilised not only for detecting current illness, but also flagging/predicting those who are likely to have mental health difficulties during their pregnancy. The decreased predictive sensitivity seen likely resulted from the fact that screening and appointments were conducted over the telephone during the pandemic, but may also be because the pandemic altered the presentation of psychiatric disorders. For example, dynamic changes to severe-acute respiratory syndrome-coronavirus-2 (SARS-CoV-2) infection rates and public health regulations could have produced stressors and mental health deterioration unforeseeable at the start of pregnancy. Mental illness may have been especially poorly anticipated and under-detected during the pandemic, leading to the observed prevalence of diagnosed perinatal mental illness not increasing during the pandemic to the level studies have indicated it should have. This is also consistent with the reported ‘prevalence-diagnosis gap’ for perinatal mental illness during the pandemic.^14^

Despite the lowered sensitivity, rates of antenatal mental health screening were high, in contrast to reports that postpartum screening rates dropped during the pandemic.^15,16^ This indicates that the pandemic's effect on screening rates likely depends on local policy and priorities. This study also observed key risk factors for perinatal psychiatric disorders, with having a psychiatric history and obesity being some of the strongest predictors; in turn, socioeconomic status/deprivation may be a key modifying factor for the effect of antenatal psychiatric illness on neonatal outcomes such as birthweight as was observed in this study. Meanwhile, the increased risk of postpartum mental illness for older mothers specifically during the pandemic may perhaps be linked to stress due to the more severe effects of SARS-CoV-2 infection on older individuals with lockdown ending, or inadequate social support considering older women are reportedly at higher risk of postpartum mental illness already.^17^

Although decreases in low birthweight and hypertensive diseases of pregnancy have previously been reported during the pandemic,^6,7^ this was not confirmed in our analyses which may be because this study was not powered to examine these outcomes. Another consideration is that we investigated women who gave birth during the second rather than the first COVID-19 lockdown. However, considering low birthweight, an already low pre-pandemic incidence may have meant rates could not decrease further during the pandemic. Meanwhile, Kasuga et al.^7^ reported zero hospitalizations due to hypertensive diseases of pregnancy during the pandemic, but considering hypertension was observed in our study, it is unclear whether their results reflected decreased hypertension incidence, severity or the impact of the pandemic on hospital admissions. The interaction effect between the pandemic and hypertension observed for birth weight implies that the pandemic-related modifications to the care of patients with hypertensive disorders of pregnancy such as those described by Jardine et al.^18^ may have been associated with some adverse impacts. Altered care could also explain the observed impact of the pandemic on Apgar scores. Mor et al.^19^ reported an increase in low 5-min Apgar scores during the pandemic which was attributed to a decline in obstetric emergency presentation resulting in delayed care, likely stemming from a fear of contracting COVID-19.

The main strength of our study was the evaluation of multiple psychiatric and psychiatric-adjacent outcomes, which provides a holistic understanding of the impact of the COVID-19 pandemic on obstetric service and health outcomes. Meanwhile, the primary limitation of this study was limited and incomplete documentation which was unavoidable due to our study design. Not all booking appointments were documented as some were out of the area, and general practice records were limited and unavailable in some mothers, relevant because some mental health episodes were documented in the primary care but not hospital records. This reinforces the idea of the difficulty health services face in detecting perinatal mental health issues. The mental health screening tool used at the booking appointment, despite being based on the Whooley questions,^8^ also involves other unique questions, such that the sensitivity and specificity may not be fully generalisable to other mental health screening tools. Further limiting generalisability is that this study was conducted in a single institution.

In conclusion, the COVID-19 pandemic required changes to healthcare provisions and may have brought about changes in perinatal mental illness risk patterns and presentation. These factors likely hindered the ability of healthcare services to detect and address psychological needs in pregnant and postpartum individuals. It may therefore be advisable to ensure routine mental health screening using a validated tool at least once more mid-pregnancy. This would be consistent with NICE advice which asks health professionals to consider screening at all antenatal appointments,^4^ but partially departs from ACOG advice where screening patients only once with a standardised tool during pregnancy could be deemed acceptable.^5^

## Supplemental Material

sj-docx-1-obm-10.1177_1753495X221139565 - Supplemental material for Impact of the COVID-19 pandemic on perinatal mental health screening, illness and pregnancy outcomes: A cohort study Click here for additional data file. Supplemental material, sj-docx-1-obm-10.1177_1753495X221139565 for Impact of the COVID-19 pandemic on perinatal mental health screening, illness and pregnancy outcomes: A cohort study by Andre C. Q. Lo, Michelle Kemp and Nikolett Kabacs in Obstetric Medicine
