# Supplementary material for: Impact of the COVID-19 pandemic on perinatal mental health screening, illness and pregnancy outcomes: A cohort study
Source: Obstet Med. 2022 Nov 28;16(3):178–83. doi: 10.1177/1753495X221139565 (PMC9708533; doi:10.1177/1753495X221139565)

**Impact of the COVID-19 pandemic on perinatal mental health screening, illness and pregnancy outcomes: a cohort study (supplementary material)**

Supplementary Appendix 1. Mental health screening tool used in booking appointments.

1. Have you been feeling down, depressed or helpless during the past month? (yes/no)
2. Have you been bothered by having little interest or pleasure in doing things this past month? (yes/no)
3. Would you like some help? (yes/no)
4. Referred to GP if yes to any of the above? (yes/no/not applicable)
5. Is anything in your life making your pregnancy difficult for you? (yes/no)
6. Comments

Supplementary Table 1. Association of covariates to incidence of psychiatric outcomes in parsimonious multivariate models.

| **Variable** | **OR (95%CI)** | | | |
| --- | --- | --- | --- | --- |
|  | **Anxiety** | | **Depression** | |
|  | **Antepartum (n=191)** | **Postpartum**  **(n=164)** | **Antepartum**  **(n=191)** | **Postpartum**  **(n=146)** |
| Pandemic | 0.90 (0.30-2.72) | 0 (0-0.03) | 1.22 (0.33-4.80) | 0 (0-0.08) |
| Age at delivery (years) | 0.88 (0.78-0.98) | 0.83 (0.64-1.05) | 0.91 (0.79-1.03) | 1.00 (0.80-1.25) |
| White ethnicity | - | - | - | 0.19 (0.02-1.38) |
| IMD (decile) | - | 0.84 (0.65-1.06) | - | - |
| Gravidity - Parity | 1.72 (0.95-3.00) | - | - | - |
| Parity | - | - | - | 2.09 (1.08-4.19) |
| Psychiatric history | 4.22 (1.43-13.64) | - | 15.01 (3.47-109.52) | 8.84 (1.84-57.51) |
| Obesity | - | 8.27 (1.35-48.80) | 7.21 (1.49-36.35) | - |
| Hypertension in pregnancy | - | 0 (0-2.15x10^44^) | - | 0 (0-8.01x10^51^) |
| Diabetes/ Gestational diabetes | - | - | - | - |
| Estimated blood loss at delivery (L) | - | - | - | 3.41 (0.70-15.60) |
| Antenatal depression | - | - | - | - |
| Antenatal anxiety | - | 8.84 (1.68-51.60) | - | 33.28 (5.38-313.81) |
| Age x pandemic (interaction) | - | 1.56 (1.15-2.21) | - | 1.46 (1.1-2.04) |

^a^Singleton births excluding terminations

Supplementary Table 2. Changes to other maternal and neonatal outcomes before and during the COVID-19 pandemic.

| **Variables** | **Pre-pandemic (n=92)** | | | **Pandemic (n=99)** | | | **p-value** | |
| --- | --- | --- | --- | --- | --- | --- | --- | --- |
|  | *n* | *Mean* | *SD* | *n* | *Mean* | *SD* | *Crude* | *Full multivariate model* |
| **Maternal** | | | | | | | |  |
| Delivery method | 67 vaginal (72.83%)  25 C-section |  |  | 70 vaginal (60.61%)  29 C-section |  |  | 0.745 | - |
| Late termination of pregnancy | 1 |  |  | 0 |  |  | - | - |
| Multiple pregnancies | 2 twin (2.17%)  90 singleton |  |  | 3 twin (3.03%)  96 singleton |  |  | >0.999 | - |
| Hypertension | 6 gestational  2 pre-eclampsia  (8.70% total) |  |  | 3 gestational  (3.03% total) |  |  | 0.093 | 0.073 |
|  |  |  |  | 2 postpartum  1 chronic |  |  | - | - |
| Gestational diabetes | 5 (5.43%) |  |  | 8 (8.08%) |  |  | 0.665 | 0.473 |
| Estimated blood loss at delivery (ml) | 92 | 538.91 | 428.23 | 96 | 564.58 | 470.75 | 0.701 | 0.465 |
| ***Neonatal****^a^* | | | | | | | |  |
| Apgar 1 min | 92 | 8.63 | 1.00 | 99 | 8.40 | 1.31 | 0.345 | 0.040 |
| Apgar 5 min | 92 | 9.78 | 0.56 | 98 | 9.68 | 0.75 | 0.539 | 0.169^b^ |
| Gestational age (days) | 85 | 278.15 | 10.12 | 97 | 277.65 | 9.83 | 0.859 | 0.683 |
| Birth weight (g) | 85 | 3425.68 | 452.05 | 97 | 3452.45 | 435.20 | 0.682 | See interaction effect |
| Birth weight category | 2 LBW (2.33%) |  |  | 1 LBW (1.01%) |  |  | 0.945 | - |
|  | 9 HBW (10.47%) |  |  | 11 HBW (11.11%) |  |  | 0.952 | - |

^a^Singleton births excluding terminations

^b^Model includes Apgar 1 min

Supplementary Figure 1. Interaction plots for effects associated with birth weight with 95% confidence intervals. Left: Higher deprivation was associated with lower birth weight in the offspring of mothers with antenatal depression. A lower Index of Multiple Deprivation (decile) indicates subjects live in lower socioeconomic areas. Right: During the pandemic, hypertensive patients had offspring with lower birthweight.


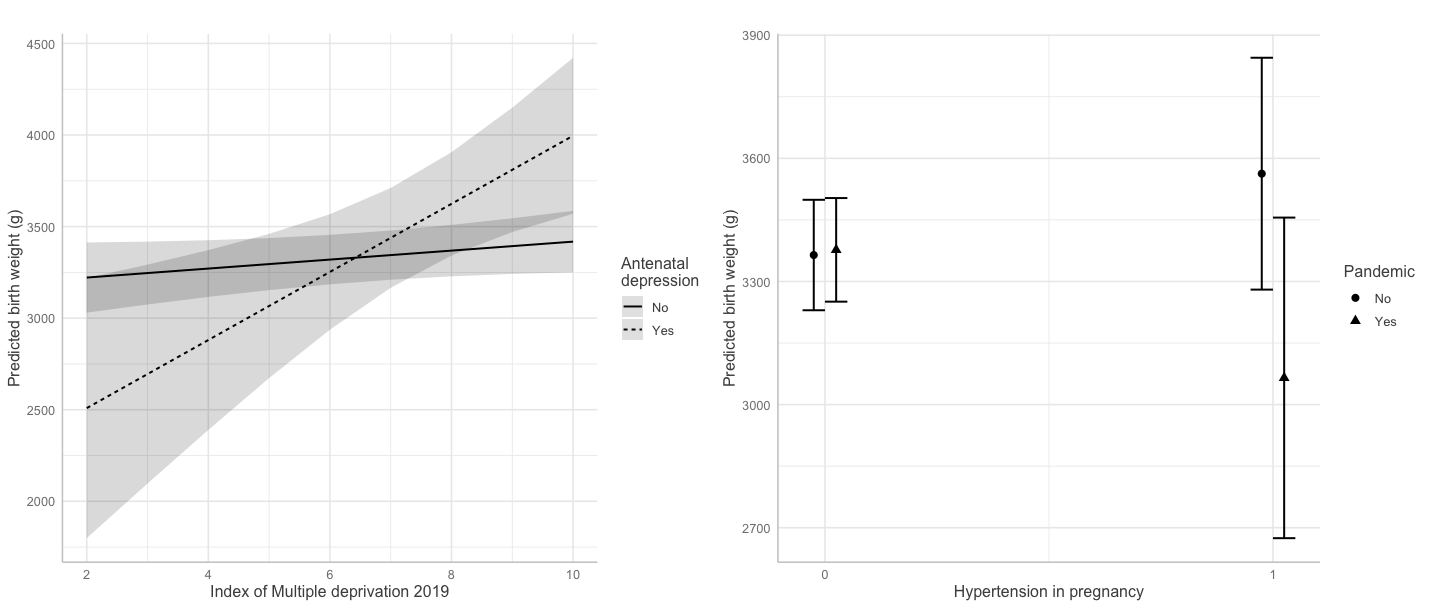

Supplement: sj-docx-1-obm-10.1177_1753495X221139565 - Supplemental material for Impact of the COVID-19 pandemic on perinatal mental health screening, illness and pregnancy outcomes: A cohort study [file sj-docx-1-obm-10.1177_1753495X221139565.docx]
